# Current phototherapy practice on Java, Indonesia

**DOI:** 10.1186/s12887-019-1552-1

**Published:** 2019-06-08

**Authors:** Mahendra T. A. Sampurna, Kinanti A. Ratnasari, Darto Saharso, Arend F. Bos, Pieter J. J. Sauer, Peter H. Dijk, Christian V. Hulzebos

**Affiliations:** 1 Department of Pediatrics, Dr. Soetomo General Hospital, Faculty of Medicine Universitas Airlangga, Surabaya, Indonesia; 2 Department of Pediatrics, Beatrix Children’s Hospital, University Medical Center Groningen, University of Groningen, Groningen, the Netherlands

**Keywords:** Curtain, Distance, Hyperbilirubinemia, Irradiance, Phototherapy

## Abstract

**Background:**

In Indonesia, the burden of severe hyperbilirubinemia is higher compared to other countries. Whether this is related to ineffective phototherapy (PT) is unknown. The aim of this study is to investigate the performance of phototherapy devices in hospitals on Java, Indonesia.

**Methods:**

In 17 hospitals we measured 77 combinations of 20 different phototherapy devices, with and without curtains drawn around the incubator/crib. With a model to mimic the silhouette of an infant, we measured the irradiance levels with an Ohmeda BiliBlanket Meter II, recorded the distance between device and model, and compared these to manufacturers’ specifications.

**Results:**

In nine hospitals the irradiance levels were less than required for standard PT: < 10 μW/cm^2^/nm and in eight hospitals irradiance failed to reach the levels for intensive phototherapy: 30 μW/cm^2^/nm. Three hospitals provided very high irradiance levels: > 50 μW/cm^2^/nm. Half of the distances between device and model were greater than recommended. Distance was inversely correlated with irradiance levels (R^2^ = 0.1838; *P* < 0.05). The effect of curtains on irradiance levels was highly variable, ranging from − 6.15 to + 15.4 μW/cm2/nm, with a mean difference (SD) of 1.82 (3.81) μW/cm2/nm (*P* = 0.486).

**Conclusions:**

In half of the hospitals that we studied on Java the levels of irradiance are too low and, in some cases, too high. Given the risks of insufficient phototherapy or adverse effects, we recommend that manufacturers provide radiometers so hospitals can optimize the performance of their phototherapy devices.

**Electronic supplementary material:**

The online version of this article (10.1186/s12887-019-1552-1) contains supplementary material, which is available to authorized users.

## Key notes

- Inappropriate phototherapy may contribute to the high burden of severe hyperbilirubinemia in Indonesia: increase the risk of bilirubin encephalopathy as well as the number of exchange transfusions.
- In half of the hospitals that we studied irradiance levels were too low, while in some the levels were very high.
- Half the distances between device and model were greater than recommended.

## Background

Worldwide, hyperbilirubinemia contributes 40 to 60% of neonatal hospital admissions [1]. Untreated hyperbilirubinemia may cause neurotoxicity that ultimately results in one of the kernicterus spectrum disorders [2]. A recent survey in Indonesia indicated a 7% incidence of hyperbilirubinemia, defined as a serum bilirubin level of > 340 μmol/L (20 mg/dL), in all newborn infants [3]. Acute bilirubin toxicity was found in 2% of newborn infants admitted [3]. Why the incidence and burden of hyperbilirubinemia is much higher in Indonesia compared to other countries is unknown.

Phototherapy (PT) has proven to be effective in reducing levels of unconjugated bilirubin. Worldwide there are many different guidelines indicating when to start and when to stop administering PT [4–6]. Studies have shown that PT can reduce total serum bilirubin (TSB) levels and thus prevent neurotoxicity and kernicterus spectrum disorders [7, 8]. For it to be effective, however, PT requires a specific level of irradiance. Studies carried out in both high-income and low-and-middle-income countries reported considerable variance in the irradiance levels of the PT devices used [9–11]. In a number of devices irradiance levels were suboptimal or insufficient, possibly causing ineffective treatment, while aggressive PT and too high levels of irradiance may be dangerous and associated with adverse effects [12, 13].

The irradiance levels of PT also depend on the distance between the PT device and the infant. All guidelines and manufacturers’ information provide recommendations concerning optimal and safe distances, but it is not clear how critical the effect of the actual distance is on irradiance levels. General practice in Indonesia is to cover the incubators or cribs with curtains when PT is administered. This is done to protect other infants in the room against the light and to increase the irradiance of the PT by reflection. Using reflective curtains may reduce TSB levels more rapidly than not using reflective curtains [5]. It is unknown, however, whether non-reflective curtains will also increase the effectiveness of PT. The aim of this study was to investigate the irradiance levels of PT in clinical practice in Indonesia. In addition, we analyzed the effect of distance between the PT device and the infant, and the effect of curtains on irradiance levels.

## Methods

Between December 2016 and August 2017 we visited 17 hospitals with Level II and Level III neonatal intensive care units (NICUs) on Java, Indonesia to measure the irradiance levels of PT devices. We had intended to study overhead as well as underneath PT devices in a clinical setting, but because we found only one underneath PT device, which was no longer in use anyway, we excluded underneath PT devices. Local NICU nurses placed a model of the silhouette of a preterm infant once in the incubator or crib and installed the overhead PT device as they would in daily practice. We had marked the model with five measurement points at 3, 12, 18, 23, and 33 cm apart, to represent an infant’s head, trunk, abdomen, knees, and feet [9]. If a device had more than one irradiation mode we measured the highest irradiance level of that PT device. The second series of irradiance measurements were done once using curtains if this was routine practice in that specific hospital. The nurses drew the curtain around the incubator or crib as they would do in everyday practice. Irradiance levels were measured in exactly the same way as without the curtains.

We measured irradiance using a new Ohmeda BiliBlanket Meter II (Konica Minolta Inc., Tokyo, Japan) that had been calibrated before use by the manufacturer. According to the specifications of the manufacturer, only one calibration per year is needed. All our measurements were done within the year of the calibration. This radiometer is designed to measure light radiation with a bandwidth range of 400 to 520 nm and peak sensitivity at 450 nm. The measuring range of the spectral irradiance is from 0.1 to 299.9 μw/cm^2^/nm. Standard PT was defined as PT with irradiance levels between 10 and 30 μw/cm^2^/nm. If irradiance levels were above 30 μw/cm^2^/nm, PT was defined as intensive PT [4].

For each PT device we used the mean values of the five replications for further calculations. Per device the means of all measurement points were averaged to one mean intensity level, in agreement with the definition of the effective surface area by the International Electrotechnical Commission and in agreement with the AAP recommendations [4].

To evaluate the effect of distance on irradiance level, we measured the distance in cm between the PT device and silhouette model at the measurement point closest to the PT device. One silhouette was used for all measurements. It is a two dimensional model with a brown color. We searched for specifications of the phototherapy devices and recommendations of the manufacturers by searching the internet using the names of the devices and the official websites of the manufacturers.

The study was approved by the Ethical Committee in Health Research of the Dr. Soetomo General Hospital Surabaya (number 390/Panke.KKE/V/2017). The need to ask Ethical approval and the need to ask consent from parents was waived by our Ethical Committee because no human subjects were involved in this study.

### Data and statistical analysis

We used SPSS for Windows, Version 21 (IBM., Corp,. Armonk, N.Y., USA) for data analysis. First, we calculated the medians and ranges of the irradiance levels for each device. Five measurements were done for each point of the silhouette. The mean of the five measurements was calculated. Next, the mean and range of the five means was used for the analysis of the total irradiance. Second, we plotted the mean irradiance levels of the PT devices of each hospital in a graph. Third, we analysed the relationship of the irradiance levels with the distance between the PT device and the model using linear regression analysis. We compared the measured irradiance levels and the distances with those stated in the manufacturers’ specifications by using independent sample T tests, and we constructed Bland-Altman graphs to analyse the differences between irradiance levels per device, with and without curtains, one for irradiance levels below 30 μw/cm^2^/nm and one for above 30 μw/cm^2^/nm. We arbitrarily chose this irradiance level to analyse whether the effect of curtains on irradiance levels depended on standard and intensive PT. We used the Mann-Whitney test to analyse the differences between irradiance levels measured with and without curtains. A *P* value below 0.05 was considered statistically significant.

## Results

We measured 77 PT devices of 20 different types in the 17 hospitals we visited. The irradiance levels of all types of devices included in this study are given in Table 1. Most types of devices used fluorescent light sources, six used light-emitting diodes (LEDs), and one used halogen. The irradiance levels of the PT devices varied widely. In three PT devices all the irradiance levels were below 10 μW/cm^2^/nm and in six devices the median levels were below 10 μW/cm^2^/nm. Eleven out of 20 PT devices had a manufacturer’s manual that contained detailed information on how to use the device and what irradiance levels the device should produce [14–24]. For the other nine devices we could not find detailed manufacturers’ specifications. Two out of 11 PT devices provided irradiance levels well below (12% or less) the given manufacturers’ specification. Six of the nine other devices with manufacturers’ specifications produced irradiance levels below the lower range of intensity recommended in the manufacturers’ specifications.

**Table 1:** Phototherapy devices per hospital, range of irradiance levels, and distances measured and recommended by the manufacturers

PT devices	No. of hospitals /devices (n/n)	Irradiance levels (μ W/cm^2^/nm)		Distance (cm)	
		Measured median (range)	Specified by MDA Manufacturer^a^	Measured median (range)	Recommended
Airshield Foto Tx System^b^	1/1	0.7 (0.5–0.9)	–	40	–
Fanem Bilitron 3006^c^ [14]	1/1	2.6 (0.7–3.6)	35–40	40	30
Tesena^b^ [15]	1/1	7.3 (5.4–7.7)	62	24	30
Choongwae PT^b^	1/ 2	7.9 (5.5–10.3)	–	41 (37–45)	–
Gammatech PT BGM^b^	1/ 4	8.3 (7–10.1)	–	50	–
My Life MP-71^b^	4/5	8.6 (6–15.5)	–	40 (40–45)	30
GEA XHZ 90^b^ [16]	7/18	11.3 (6.6–39.4)	9–27	40 (25–59)	–
Nidea PT 2000–1600^b^	1/ 2	11.7 (7.1–16.1)	–	30	–
Medela^b^ [17]	4/12	13.3 (3.5–38.5)	14–31	30 (28–40)	25–40
YON DON PT^b^	1/ 2	13.7 (9.1–19.7)	–	40	–
Onemed GLQ 2^b^	1/1	14.7 (13–15.7)	–	40	–
Ohmeda 2 pcs PT Light II Halogen + Philips Blue^d^ [18]	1/1	15.9 (8.4–27.8)	3–76	(15–35)	40
Draeger PT 4000^b^ [19]	2/3	19.1 (9.7–35)	14–27	32.5 (30–35)	30–40
GE Lullaby Fluoresens^b^ [20]	1/ 2	23.3 (13.1–25)	20–30	35	30
Philips TL 20 W/52 SLV/25^b^	3/5	30.9 (9.1–36.8)	–	35 (29–37)	–
Bistos BT 400^c^ [21]	2/3	32.7 (11.9–79.3)	> 30	42 (34–50)	30
GE Lullaby LED^c^ [22]	4/9	39.9 (12.5–57.9)	22–45	40 (32–45)	30
Tende LED^c^ [23]	2/ 2	46 (36.5–127)	33–120	32.5 (30–35)	40
Seefar 4000 Spot^c^	1/ 2	65.2 (44.4–80.6)	–	25	–
Novos Bilisphere LED^c^ [24]	1/1	91.4 (65.3–95.7)	> 60	17	20

^a^Data provided by the Medical Device Agency (MDA) or Manufacturers in μ W/cm^2^/nm, the lowest and highest value are presented [14–24]

Type of PT device: ^b^Fluorescent, ^c^LED, ^d^Halogen

**Fig. 1:**
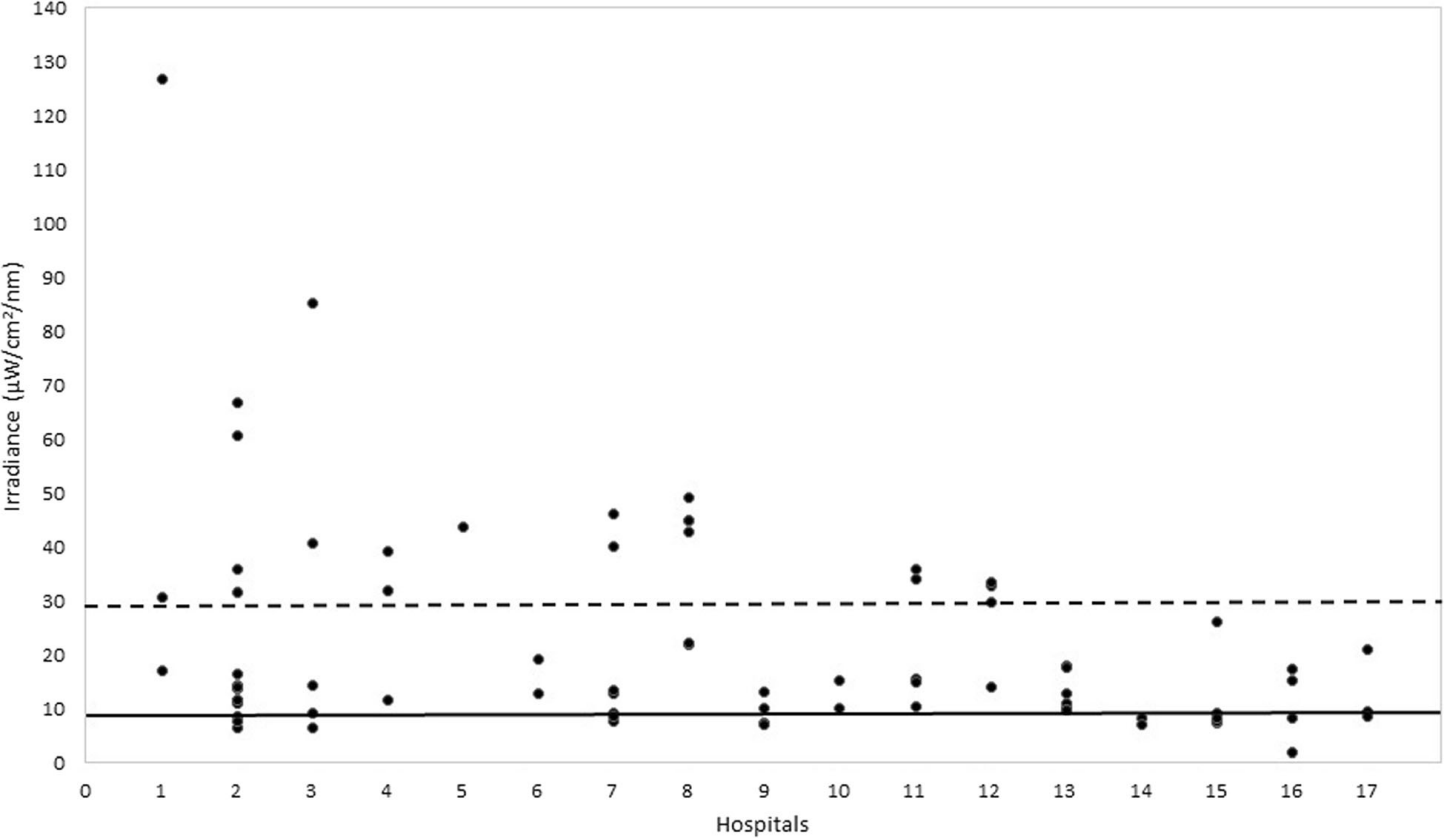
Mean irradiance levels of all phototherapy devices for each of the 17 hospitals on Java, Indonesia. The unbroken line in the graph indicates the level above which standard phototherapy is effective (10 μW/cm^2^/nm) and the broken line indicates the level above which phototherapy is considered intensive PT (30 μW/cm^2^/nm)

Regarding the hospitals, we found a wide variation in the irradiance levels, both between and within hospitals. Figure 1 shows the mean irradiance levels of all PT devices in all hospitals. The irradiance levels ranged from 0.68 to 127 μW/cm^2^/nm. Nine (53%) of the hospitals used PT devices with an intensity below the standard PT of 10 μ W/cm^2^/nm. Eight (47%) of the hospitals did not have PT devices that produced an intensity of at least 30 μ W/cm^2^/nm, the level required for intensive PT. Three hospitals used PT devices with irradiance levels above 50 μ W/cm^2^/nm. These were mostly LED-based PT devices. The irradiance level of one LED device was too low.

**Fig. 2:**
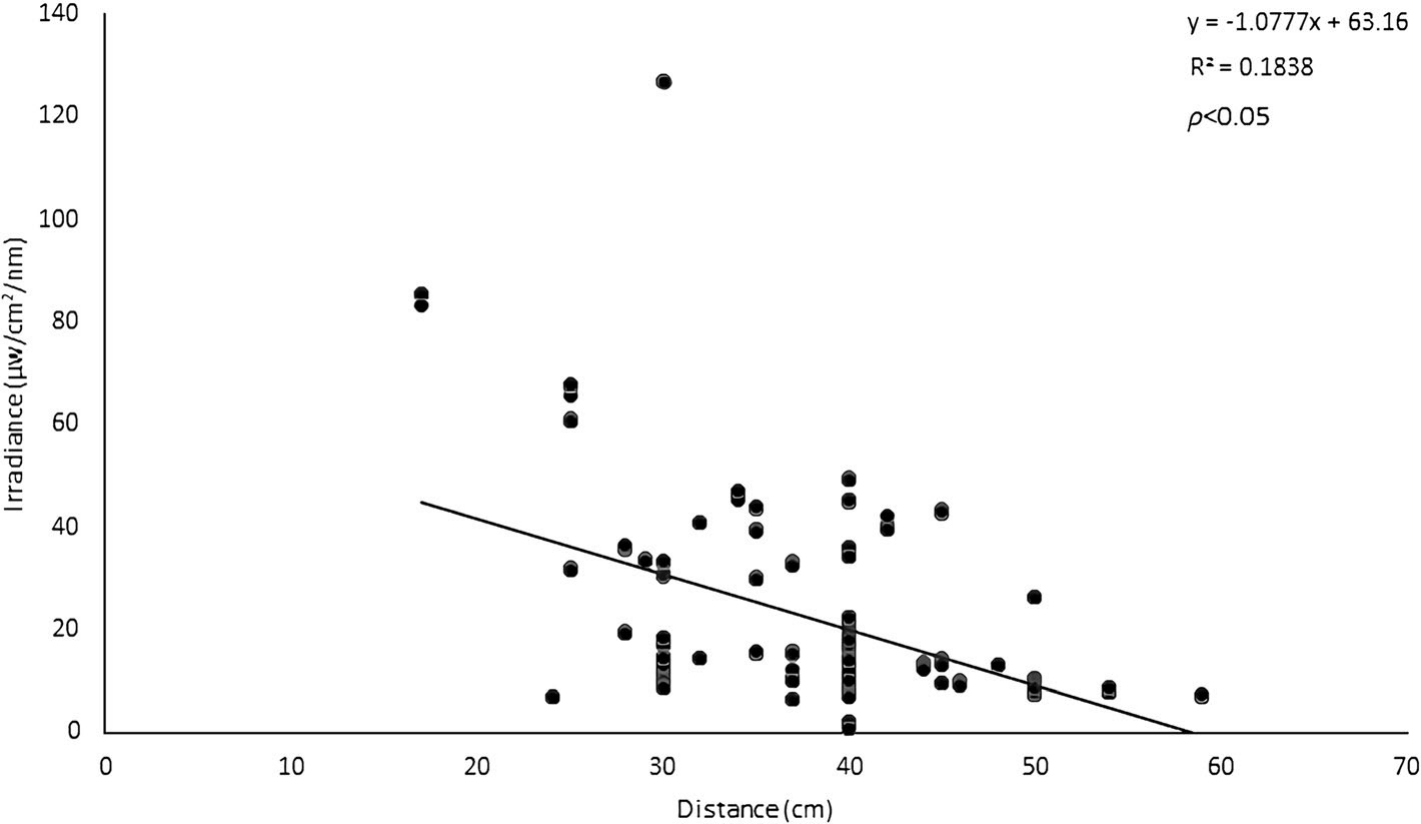
The relationship between the distance of the phototherapy device and the model and the mean irradiance levels of each device, measured without curtains. For the irradiance levels we calculated the mean of the measurements of the five points marked on the model (see Methods). The line in the graph represents the regression line, the concomitant formula is presented in the upper right-hand corner of the graph

The distance between the model and PT devices ranged from 17 to 59 cm (mean ± SD: 37.8 ± 8.3 cm), while the manufacturers recommended distances ranging from 20 to 40 cm (mean ± SD: 31.9 ± 6.3 cm) that, with a difference of 5.9 cm, is significantly closer (*P* < 0.026). Table 1 also provides the distance between the PT devices and the infant as recommended by the manufacturers and the distance used in daily practice. We found one half (20 out of 40) of the distances between device and model to be greater than the distance recommended by the manufacturers. As we show in Fig. 2, there was a significant inverse relationship between the distance of the PT device to the model and the irradiance levels, (R^2^ = 0.1838; *P* < 0.05). With every 1 cm increase in distance, intensity decreased by approximately 1.1 μ W/cm^2^/nm. Thus, the mean difference between the measured and recommended distance of 5.9 cm accounts for a mean reduction in intensity of 5.9 times 1.0777 equals 6.4 μ W/cm^2^/nm.

**Fig. 3:**
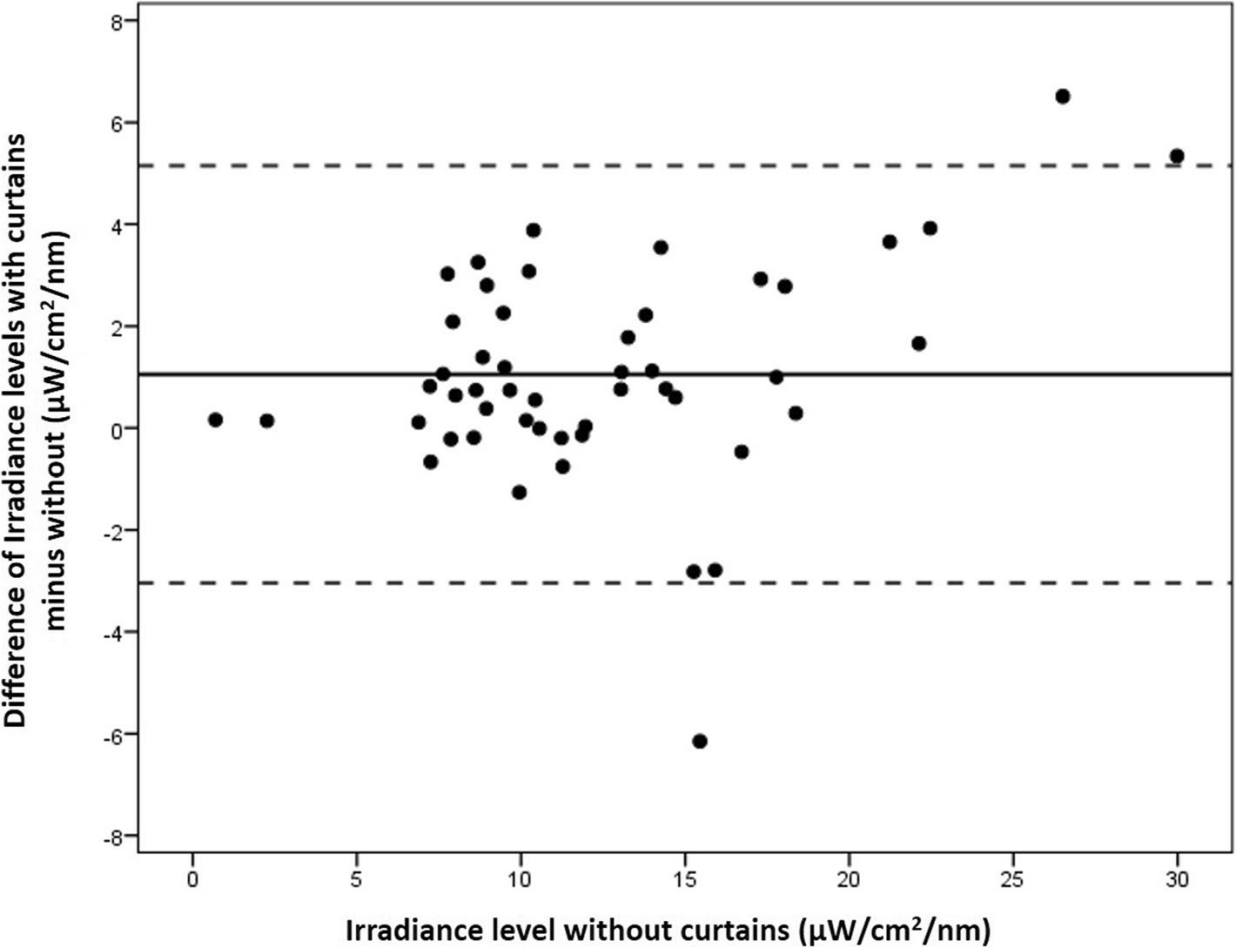
Bland-Altman plot showing the difference of irradiance levels of each phototherapy device, with curtains compared to without curtains, for those phototherapy devices that had irradiance levels below 30 μw/cm^2^/nm. Each phototherapy device presents one dot, the mean (SD) difference was 1.05 (2.08) μW/cm^2^/nm higher when using curtains (*P* = 0.370)

**Fig. 4:**
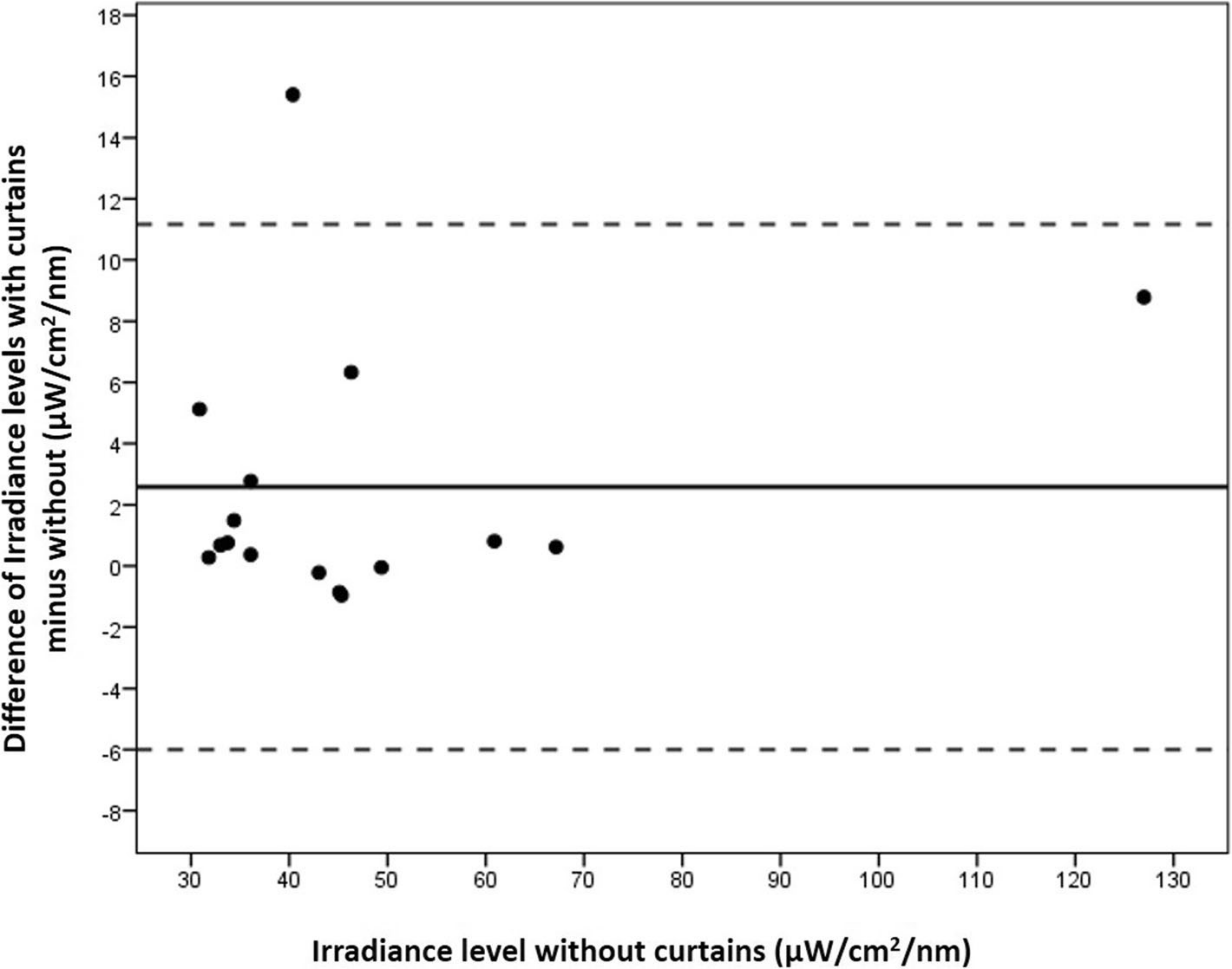
Bland-Altman plot showing the difference of irradiance levels of phototherapy with curtains compared to without curtains, for phototherapy devices that have irradiance levels above 30 μw/cm^2^/nm. Each phototherapy device presents one dot. The mean (SD) difference was 2.58 (4.37) μW/cm^2^/nm higher when using curtains (*P* = 0.572)

One hospital did not use curtains, eight hospitals used white curtains, and one hospital used black curtains, the remaining seven hospitals used a variety of mostly light-coloured curtains. The difference in irradiance levels, with and without curtains, was highly variable and ranged from − 6.15 and + 15.4 μW/cm2/nm per measurement point, with a mean difference (SD) of 1.82 (3.81) μW/cm2/nm, and the overall effect was not significant (*P* = 0.486). In Fig. 3 we present the difference in irradiation levels per device, with and without curtains, for standard PT with irradiance levels below 30 μ W/cm^2^/nm, and in Fig. 4 for intensive PT with irradiance levels above 30 μ W/cm^2^/nm. For irradiance levels below 30 μ W/cm^2^/nm, the mean (SD) difference was 1.05 (2.08) μ W/cm^2^/nm higher with curtains (*P* = 0.370). Above 30 μ W/cm^2^/nm, the mean (SD) difference for irradiation levels was 2.58 (4.37) μ W/cm^2^/nm higher with curtains, but it also failed to reach statistical significance (*P* = 0.572). The raw data of PT irradiance from all hospital is provided in Additional file 1.

## Discussion

In this observational study we found that the intensity of phototherapy varied considerably both within and between hospitals on Java, in Indonesia. Devices with irradiance levels below which PT is recommended were in use in more than half of the hospitals. Moreover, nearly half of the hospitals did not have devices that provided sufficient irradiance for intensive PT. By contrast, some hospitals had PT devices that produced very high irradiance levels that perhaps enhanced the efficacy of PT [25, 26], but were perhaps not always safe [12, 13]. Altogether, the effect of PT on hyperbilirubinemia may be highly unpredictable.

The irradiance levels of the PT devices varied considerably, with a high percentage of ineffective standard and intensive PT. This finding was in line with other studies performed in either developed or developing countries [9–11]. According to Bhutani and colleagues, there are four different explanations for low irradiance levels of PT in developing countries [27]. First, the sources of light may be inferior. Second, maintenance constraints may play a role. Third, countries may lack a steady supply of electrical power. Lastly, awareness of the requirements for effective phototherapy may be lacking, such as the importance of the distance between the infant and the PT device.

The most likely explanations for the low levels of irradiance found on Java are inferior PT devices, lack of awareness of the requirements for effective PT, insufficient maintenance, and too great a distance between the infant and the PT device. Increasing awareness of the factors that affect PT efficacy will undoubtedly result in better performance of the devices and smaller distances [28]. The AAP recommends measuring the intensity of PT devices regularly [4]. On Java, however, none of the hospitals we visited had a radiometer to measure irradiance. Neither did any of the companies that supplied the PT devices to the hospitals also provide radiometers. We do not know whether this is due to a lack of awareness on the part of the suppliers of PT devices or on the part of those who purchase PT devices, or whether other constraints play a role. Furthermore, it is common knowledge that irradiance levels of conventional lamps and tubes decrease with use. These lamps and tubes should therefore be checked periodically and replaced if necessary. In most hospitals on Java, however, it is not customary to replace PT lamps that still seem to be working properly. On the other hand, it is not possible to check whether a PT device is functioning properly without a radiometer. Our results are in line with data from Nigeria, where routine irradiance monitoring is also uncommon, whereas periodically monitoring of irradiance is recommended [29, 30].

The majority of PT devices with low irradiance were fluorescent tube-based devices, but there was one LED-based PT device that had low irradiance. Olusanya and colleagues found that irradiance decay was higher and occurred faster in fluorescent tube-based PT devices compared to LED-based PT devices [29]. LED-based PT devices also show irradiance decay, but after a much longer period of use. LED-based PT devices may last up to 20,000 h while fluorescent tube-based PT devices may require lamp replacement every 3000 h [27]. Therefore, LED-based PT should be preferred in resource-constrained settings. Nevertheless, regular irradiance monitoring is always essential, also in resource-constrained settings [4, 30].

Only for 11 of the 20 devices used, did we find the manufacturers’ specifications. This is odd, because without these specifications healthcare workers cannot be expected to know the performance of the device, the recommendations for effective use and, even more essential, the safety precautions. On Java, all hospitals have sufficient electrical power supplies, so the third explanation of Bhutani and colleagues [27] does not apply. Finally, we often found that the distances between the model and the PT device, as installed by the nurses according to their routine clinical practices, were too great and often not in accordance with the manufacturers’ recommendations. Significant improvements in irradiance could be gained if all parties involved in selling, buying, and using PT devices were better informed about the principles of effective PT.

In a few instances, we also found very high irradiance levels - up to 127 μ W/cm^2^/nm. This was the case in specific devices that delivered PT to the infant in 360 degrees and in some overhead PT devices using blue light LEDs. Levels above 50 μ W/cm^2^/nm are more effective in decreasing TSB levels faster [25, 26]. Nevertheless, several studies reported that intensive phototherapy may cause deoxyribonucleic acid (DNA) damage and increased apoptosis [31–33]. One epidemiologic study reported that infants with a history of phototherapy the risk of cancer is up to two times higher than in non-exposed infants, albeit a very low absolute risk [34]. Other studies reported a tendency towards increased mortality, especially in extremely low birth weight infants, which was not related to PT intensity, but to the duration of PT [12, 13]. To prevent potential adverse effects it is logical to limit the duration of PT. In addition, an effective but short period of PT will interfere less with parent- child interaction.

We found no overall significant effect on irradiance levels in the case of curtains drawn around the incubator or crib. In most hospitals on Java, curtains are used around cribs with a PT device to protect other infants against the light and to increase the irradiance level of PT devices by reflection at the same time. Eggert and colleagues suggested lining the incubator with reflective cloth to increase the radiant power of PT devices [35]. This study was supported by studies in Germany, India, and Malaysia that reported that a white curtain around the PT device may reduce TSB faster, without reducing the total time of administering PT [36–38]. The National Institute for Health and Care Excellence (NICE) guideline does not recommend using curtains because it prevents observing the baby [5]. As most of the curtains used in our study were light-coloured (but not white and possible not reflective), we believe that this may be the explanation for the unpredictable effect we found. In fact, this observation underlines the need to measure irradiance. A reason for the rather wide variation between the measurements with and without curtains might be that we studied curtains made of different materials and with different colors. Another study is needed to evaluate effects of different reflective materials and of different colors on irradiance.

Indonesia has not yet a national guideline on the management of hyperbilirubinemia and no specific recommendation for phototherapy. Pediatricians in Indonesia are advised to use the AAP guidelines for the treatment of hyperbilirubinemia. Previously, we found that only 54% of pediatricians in Indonesia follow these guidelines [39]. We think that a national guideline should contain recommendations and information about phototherapy that supports a better awareness of the requirements for effective phototherapy.

Our study has some limitations. First, we included only 17 hospitals on Java. This limits the generalizability of our findings, because these 17 hospitals may not be representative of the whole of Indonesia. We suspect that other hospitals in other parts of the country are perhaps less well-equipped. Our results could therefore be an overestimation of the effectiveness of PT devices for the whole country. Second, we used a single radiometer. The values we found might have been different had we used device-specific radiometers [40]. The advantage of the radiometer we used is that it covered a wide range of wavelengths. Third, the model we used is not a real infant. Nevertheless, we believe that the model approximated irradiance levels quite well because we measured at five points. Fourth, some of the PT devices in the hospitals had been in use for quite some time. We were not able to retrieve the manufacturer’s information and specifications for nine of the 20 devices.

Our findings may have implications for clinical practice. We recommend measuring the irradiance levels of all PT devices in all hospitals in order to provide effective standard and intensive PT. Manufacturers should provide essential information about the performance and safety of their devices, in addition they should provide radiometers to measure irradiance levels, and the hospitals should buy them and use them. Significant improvements in irradiance would be gained if all who sell, buy, and use PT devices were better informed about the principles of effective PT.

## Conclusion

Half of the hospitals that we studied on Java, Indonesia, use PT devices that provide PT levels that are too low, while some provide very high levels of PT. Given the risks of either insufficient PT or adverse effects, we recommend that manufacturers provide radiometers and that heath care providers measure irradiance to optimize the performance of their PT devices so as to reduce the burden of severe hyperbilirubinemia in Indonesia.

## Additional file

Additional file 1: Intensity data of all phototherapy devices from participating hospitals. (XLSX 86 kb)

## Data Availability

The datasets used and/or analysed during the current study available as a supplementary file.

## Abbreviations

AAP: American Academy of Pediatrics
LED: Light-emitting diode
NICE: The National Institute for Health and Care Excellence
NICU: Neonatal intensive care units
PT: Phototherapy
TSB: Total serum bilirubin
